# Sensitivity to Change and Minimal Important Differences of the LupusQoL in Patients With Systemic Lupus Erythematosus

**DOI:** 10.1002/acr.22850

**Published:** 2016-09-02

**Authors:** Kathleen McElhone, Janice Abbott, Chris Sutton, Montana Mullen, Peter Lanyon, Anisur Rahman, Chee‐Seng Yee, Mohammed Akil, Ian N. Bruce, Yasmeen Ahmad, Caroline Gordon, Lee‐Suan Teh

**Affiliations:** ^1^ Royal Blackburn HospitalBlackburnUK; ^2^ University of Central LancashirePrestonUK; ^3^ Queen's Medical Centre Campus, Nottingham University HospitalsNottinghamUK; ^4^ University College LondonLondonUK; ^5^ Doncaster Royal InfirmaryDoncasterUK; ^6^ Royal Hallamshire HospitalSheffieldUK; ^7^ The Kellgren Centre for Rheumatology, National Institute for Health Research Manchester Musculoskeletal Biomedical Research Unit, Central Manchester University Hospitals National Health Service Foundation Trust and Arthritis Research UK Centre for Epidemiology, Centre for Musculoskeletal Research, Institute of Inflammation and Repair, University of Manchester, Manchester Academic Health Science CentreManchesterUK; ^8^ Peter Maddison Rheumatology Centre, Betsi Cadwaladr University Health Board, Llandudno HospitalLlandudnoUK; ^9^ University of Birmingham, National Institute for Health Research Wellcome Trust Clinical Research Facility, University Hospital Birmingham National Health Service Foundation Trust, and West Birmingham Hospitals National Health Service TrustBirminghamUK

## Abstract

**Objective:**

As a health‐related quality of life (HRQOL) measure, the LupusQoL is a reliable and valid measure for adults with systemic lupus erythematosus (SLE). This study evaluates the responsiveness and minimal important differences (MIDs) for the 8 LupusQoL domains.

**Methods:**

Patients experiencing a flare were recruited from 9 UK centers. At each of the 10 monthly visits, HRQOL (LupusQoL, Short Form 36 health survey [SF‐36]), global rating of change (GRC), and disease activity using the British Isles Lupus Assessment Group 2004 index were assessed. The responsiveness of the LupusQoL and the SF‐36 was evaluated primarily when patients reported an improvement or deterioration on the GRC scale and additionally with changes in physician‐reported disease activity. MIDs were estimated as mean changes when minimal change was reported on the GRC scale.

**Results:**

A total of 101 patients were recruited. For all LupusQoL domains, mean HRQOL worsened when patients reported deterioration and improved when patients reported an improvement in GRC; SF‐36 domains showed comparable responsiveness. Improvement in some domains of the LupusQoL/SF‐36 was observed with a decrease in disease activity, but when disease activity worsened, there was no significant change. LupusQoL MID estimates for deterioration ranged from −2.4 to −8.7, and for improvement from 3.5 to 7.3; for the SF‐36, the same MID estimates were −2.0 to −11.1 and 2.8 to 10.9, respectively.

**Conclusion:**

All LupusQoL domains are sensitive to change with patient‐reported deterioration or improvement in health status. For disease activity, some LupusQoL domains showed responsiveness when there was improvement but none for deterioration. LupusQoL items were derived from SLE patients and provide the advantage of disease‐specific domains, important to the patients, not captured by the SF‐36.

## INTRODUCTION

The survival of patients with systemic lupus erythematosus (SLE) has improved in the last 50 years from less than 50% at 5 years in 1955 to 85% at 10 years and recently, 75% at 20 years 1. The Outcome Measures in Rheumatology Clinical Trials group and the US Food and Drug Administration (FDA) have recommended that for clinical trials and observational studies, health‐related quality of life (HRQOL) should be assessed using both generic and disease‐specific measures, allowing comparison with healthy samples, estimates of health utilities, and disease‐specific information known to be important to patients 2, 3. HRQOL instruments provide a standardized, valid, and reliable way of gaining the patient's perspective as to how they are affected by SLE and the benefits and limitations of interventions. HRQOL in SLE is poorly correlated with the clinicians' assessment of disease activity and damage 4, 5, as some symptoms are only known to the patient (e.g., fatigue, nausea). Therefore, HRQOL measurement can provide added value because it can supply information not captured by other outcome measures. HRQOL may be informative not only as an efficacy measure, but it also potentially reflects safety issues, and for these reasons HRQOL is becoming important in labeling claims 6, 7.

Box 1 **Significance & Innovations**

- The LupusQoL, a patient‐derived disease‐specific health‐related quality of life (HRQOL) measure for adults is sensitive to change in health status and can be recommended for use in clinical trials.
- The LupusQoL domain minimal important differences for deterioration range from −2.4 to −8.7 and for improvement from 3.5 to 7.3.
- LupusQoL items were derived from systemic lupus erythematosus patients and provide the advantage of disease‐specific domains, important to them, not captured by the Short Form 36 health survey.
- These results will allow appropriate power calculations and interpretation of HRQOL measurements in clinical trials and longitudinal observational studies.

The LupusQoL is a valid, reliable, patient‐derived, disease‐specific HRQOL measure for adults with SLE 8 that contains items/domains more relevant to patients with SLE than generic measures 9. As with many HRQOL measures, the interpretation of the data may be problematic and should not be based solely on *P* values, especially if HRQOL is a secondary outcome when a trial tends not to be powered for HRQOL. To aid the interpretation of the LupusQoL, evaluation is required to assess its sensitivity to change (the ability to detect an improvement or deterioration when patients deem themselves to have improved or deteriorated) 10 as advocated by the regulatory bodies 2, and to estimate the minimal important difference (MID), the smallest difference that patients perceive as beneficial or harmful 11.

This study aimed to evaluate these parameters, using both anchor‐based and distribution‐based methods, for each domain of the LupusQoL and the Short Form 36 health survey (SF‐36). Specifically, the study looked at the ability of the scales to detect an improvement in HRQOL following treatment of a severe or moderate flare, to detect deterioration in HRQOL (e.g., when patients fail to have their disease controlled by their initial treatment plan), and to estimate the MIDs. The responsiveness of the LupusQoL and the SF‐36 was evaluated primarily when patients reported an improvement or deterioration on the global rating of change (GRC) scale 12 and, second, with changes in physician‐reported disease activity.

## PATIENTS AND METHODS

#### Study design

This was a prospective, longitudinal, observational study. The study was granted multicenter Research Ethics Committee approval (MREC 02/05/035) and was carried out in compliance with the Helsinki Declaration at the following rheumatology units: Bangor, Birmingham (2 centers), Blackburn, University College London, Nottingham, Manchester, Doncaster, and Sheffield. All patients gave written informed consent.

#### Patient inclusion and exclusion

Patients were recruited over an 18‐month period and were followed at 4‐week (± 2 weeks) intervals for 9 months. The inclusion criteria were fulfillment of ≥4 American College of Rheumatology (ACR) criteria for SLE 13, 14, ages ≥16 years, literate ability in the English language, willingness to give written informed consent, and a flare of SLE requiring specific treatment. A flare was defined as a significant increase in disease activity, resulting in a British Isles Lupus Assessment Group 2004 (BILAG‐2004) index A or B score, based on criteria that are new or worse 15, 16, 17. To be included in this study, patients had to require an increase in therapy defined as ≥1 of the following: an increase of oral prednisolone to ≥20 mg/day, introduction of methotrexate, parenteral methylprednisolone, and/or other immunosuppressive therapy (e.g., cyclophosphamide, rituximab). The exclusion criteria were ages <16 years, inability to read English, inability to give valid consent, and pregnancy.

#### Assessment measures

Demographic and clinical details were recorded at baseline by the clinician (date of birth, sex, date of diagnosis, fulfilment of ACR criteria for SLE, ethnic group 18, marital status, and current therapy). The Systemic Lupus International Collaborating Clinics/ACR Damage Index (SDI) 19 was reported twice, at baseline and at the end of the study. The BILAG‐2004 disease activity index was assessed at each visit.

The original English version of the LupusQoL (4‐week recall period) 8 was completed by the participant at each time point. It has 8 domains: physical health, pain, planning, body image, burden to others, intimate relationships, emotional health, and fatigue. This instrument has good internal reliability (Cronbach's α = 0.88–0.96), test–retest reliability (intraclass correlation coefficient [ICC] 0.72–0.93), and concurrent validity with comparable domains of the SF‐36 (ICC 0.71–0.79). It has acceptable ceiling effects and minimal floor effects. Scoring of each domain of the LupusQoL is such that 0 = worst health and 100 = best health 8.

Patients completed the SF‐36 (UK version 1) with a 4‐week recall at each assessment 20. The SF‐36 measures 8 dimensions of health: physical functioning, social functioning, role limitations due to physical problems, role limitations due to emotional problems, mental health, energy/vitality, bodily pain, and general health perception. Domain scores can range from 0 to 100 (higher scores indicate a better HRQOL).

To estimate patient‐reported change, each domain of the LupusQoL and the SF‐36 incorporated the GRC scale 12. Patients were asked to rate change in each domain over the past 4 weeks from 7 (a very great deal better) to −7 (a very great deal worse) with 0 indicating no change. Scores of −1 to 1 were classified as no change, with −7 to −2 as deterioration and 2 to 7 as improvement. Within the deterioration and improvement categories, scores of 2, 3, −2, and −3 were considered to represent minimal, but nevertheless, important changes.

At baseline and each review visit, the clinician assessed the SLE disease activity using the BILAG‐2004 index 15. The BILAG‐2004 category scores A to E are based on intention to treat, where A = severe disease activity, B = moderate disease activity, C = mild, stable disease, D = inactive disease but previously affected system, and E = a system that has never been involved. Changes in overall disease activity between consecutive time points, as measured by the BILAG‐2004 index, were defined as follows: 1) deterioration, with any system changing to A from B/C/D or to B from C/D 21; 2) improvement, with all systems changing from A to B/C/D and B scores changing to C/D 22, with no deterioration in any system (one persistent B score was allowed if there was improvement from A or B in at least 1 other system); 3) persistent inactive disease, with all systems scored as C/D/E at both time points; and 4) persistent active disease, with A or B system scores remaining unchanged but without overall improvement or deterioration. When changes of activity of a single BILAG system were analyzed, the above definitions applied, but only for that system, and no persistent B score was allowable for improvement.

#### Statistical methods

The sample size calculation was based on summary statistics from previous work during the LupusQoL development and based on the changes of the physical health domain. A sample size of 52 would have 80% power to detect a difference in means of 4, assuming an SD of 10, using a paired *t*‐test with a 5% significance level. The intention was to recruit 104 patients, to allow for patients who did not report changes in HRQOL, expected smaller effect size for the other domains, missing data, and dropouts. All analyses were performed using Stata Release software, version 13 23.

#### Determination of sensitivity to change (responsiveness)

The primary method for assessing responsiveness was based on patient‐reported GRC scores. Responsiveness was also examined using physician‐reported disease activity change scores. Based on GRC or disease activity change scores, each domain of both HRQOL measures was evaluated to determine its ability to detect an improvement in HRQOL following treatment of a flare and to detect deterioration in HRQOL (e.g., when treatment has undesirable and troublesome side effects or the patients fail to have their disease controlled by their initial treatment plan). Responsiveness was estimated as the mean change in HRQOL domain score across participant‐reported improvements or improvement of disease activity, and across participant‐reported deteriorations or deterioration of disease activity, between consecutive assessments. We calculated 95% confidence intervals (95% CIs) using robust methods for estimating the standard error in Stata, based on the approach proposed by Huber 24.

Additionally, standardized response means (SRMs), the ratio of the mean change of the domain score between consecutive observations and the corresponding estimated SD of the change score, were reported based on GRC scores. Effect sizes, for which the mean change of each domain score was standardized using the estimated SD of the baseline score, were also reported based on GRC scores. Both are standardized measures of responsiveness, with the SRM having the advantage that it is less affected by the heterogeneity of the sample by using a more appropriate SD, namely that of the change score. SRMs or effect sizes of 0.2, 0.5, and 0.8 are deemed to demonstrate small, moderate, or large responsiveness, respectively 25.

We also explored changes in relevant domains of the LupusQoL and SF‐36 to changes of key systems in the BILAG‐2004 index. The musculoskeletal and mucocutaneous systems are the most commonly affected systems in SLE patients, and therefore we explored the relationships between musculoskeletal system changes and the changes in physical health and pain domains of the LupusQoL and the changes in physical functioning and bodily pain domains of the SF‐36. We also explored the mucocutaneous system changes and the changes in the body image domain of the LupusQoL.

#### Estimation of MIDs

Methods for estimating the MID are either anchor‐based (sometimes referred to as minimum clinically important difference) or distribution‐based. We use the term MID as this is the more dominant term in the current literature 26 for both approaches, as ultimately they seek to establish the same property. We will illustrate the difference in the methodology by using MID(a) for the anchor‐based approach or MID(d) for the distribution‐based approach. No single approach is perfect, and multiple strategies are likely to enhance the interpretability of changes in HRQOL scales 11, 27. An anchor‐based method was used as the primary approach (as preferred by the FDA) 2, based on the average change in LupusQoL or SF‐36 scores for the subset of patients who were considered to have a small but discernible change in that particular HRQOL domain. These analyses were complemented by distribution‐based approaches based around the common standards of 1 SEM, using data from McElhone et al 8 and 0.5 SD, which corresponds to a medium effect 28, 29.

## RESULTS

#### Patient recruitment and followup

**Figure 1:**
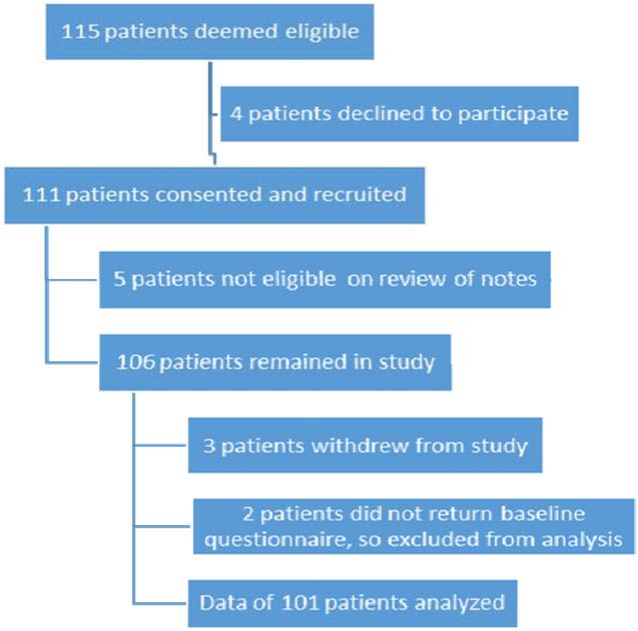
Flow diagram of patient progress through study procedures.

During the 18‐month recruitment period, a total of 115 patients from 9 centers were deemed eligible for the study and approached. Four patients declined to participate and 111 patients were recruited. Of these, 101 patients completed the study and are reported here (Figure 1).

#### Patient demographics, disease activity, and damage

The baseline demographic and clinical characteristics and HRQOL of the patients are provided in Tables 1 and 2. There were 42 A flares in 41 patients (one patient had A flares in both musculoskeletal and mucocutaneous systems) and 130 B flares, with some patients having both A and B flares. The frequency of flares in the different BILAG‐2004 systems is shown in Supplementary Table 1 (available on the *Arthritis Care & Research* web site at http://onlinelibrary.wiley.com/doi/10.1002/acr.22850/abstract). Only 28% of the patients scored ≥1 for damage on the SDI (Table 2).

**Table 1:** Patient baseline demographic and clinical characteristics^a^

Female	95 (94)
Age, mean ± SD years	40.9 ± 12.8
Disease duration, mean ± SD years	9.3 ± 8.1
Ethnic distribution (n = 99)	
White (British, Irish)	62 (63)
Black (Caribbean, African)	12 (12)
Asian (Indian, Pakistani, Bangladeshi)	14 (14)
Chinese	1 (1)
Other Asian	3 (3)
Mixed	7 (7)
Clinical characteristics (ACR criteria)	
Malar rash	43 (43)
Photosensitivity rash	47 (47)
Discoid rash	12 (12)
Mouth ulcers	47 (47)
Arthritis	92 (91)
Serositis	45 (45)
Renal disease	21 (21)
Central nervous system disease	8 (8)
Hematologic disease	73 (72)
Positive antinuclear antibodies	96 (95)
Positive dsDNA, Sm, or APA antibodies	80 (79)
Marital status (n = 94)	
Married	53 (56)
Single	36 (38)
Divorced	5 (5)

a Values are the number (%) unless stated otherwise. ACR = American College of Rheumatology; dsDNA = double‐stranded DNA.

**Table 2:** Patient baseline disease activity, damage, and health‐related quality of life^a^

BILAG‐2004 index	16.4 ± 8.1
SLICC/ACR DI (n = 98)	0.56 ± 1.18
SLICC/ACR DI, no. (%)	
Score of 0	71 (72)
Score of 1	14 (14)
Score of 2	6 (6)
Score of ≥3	7 (7)
LupusQoL domain scores	
Physical health	42.6 ± 26.6
Pain	40.9 ± 28.0
Planning	46.4 ± 32.2
Intimate relationships	37.8 ± 33.5
Burden to others	33.3 ± 28.2
Emotional health	51.4 ± 25.8
Body image	45.5 ± 29.1
Fatigue	34.1 ± 26.0
SF‐36 domain scores	
Physical functioning	38.1 ± 26.9
Bodily pain	31.8 ± 23.1
Social functioning	42.3 ± 29.7
General health perceptions	25.0 ± 18.6
Role emotional	28.7 ± 39.4
Mental health	51.0 ± 21.5
Role physical	12.1 ± 27.5
Vitality	21.9 ± 20.0

a Values are mean ± SD unless indicated otherwise. BILAG‐2004 = British Isles Lupus Assessment Group 2004; SLICC = Systemic Lupus International Collaborating Clinics; ACR = American College of Rheumatology; DI = damage index; SF‐36 = Short Form 36 health survey.

#### HRQOL: LupusQoL and SF‐36 at baseline (flare)

HRQOL was severely impaired at baseline, with mean LupusQoL scores ranging from 33.3 (burden to others) to 51.4 (emotional health), while mean SF‐36 domain scores ranged from 12.1 (role physical) to 51.0 (mental health) (Table 2). Scores for comparable LupusQoL/SF‐36 domains were highly correlated (pain/bodily pain r = 0.76; physical health/physical function r = 0.82; emotional health/mental health r = 0.74; fatigue/vitality r = 0.66), although observed mean scores were consistently higher for LupusQoL than for SF‐36 domains (Table 2).

#### Sensitivity to change (responsiveness)

There were 911 (90.2%) completed HRQOL questionnaires across the 10 time points. The amount of complete change score data, however, varied across HRQOL domains and time points. For the LupusQoL, 6 of the 8 domains had >83% of change score data available, ranging from 776 (85.4%) for physical health to 757 (83.3%) for fatigue, with the other domains having lower percentages of change score data available (body image 616 [67.8%] and intimate relationships 541 [59.5%]). For the SF‐36 the amount of change score data available ranged from 773 (85.0%) for general health to 761 (83.7%) for role physical.

All domains of the LupusQoL worsened significantly when patients reported a deterioration of their health status, and all domains improved significantly when patients reported an improvement in their health status (Tables 3 and 4). There was little change in the mean LupusQoL scores when patients reported no change in their health status (1, 0, and −1 on the GRC score). Mean change scores ranged from 0.6 (95% CI −0.4, 1.6) in the physical health domain to 2.5 (95% CI 1.2, 3.8) in the burden to others domain. For deterioration, the mean LupusQoL change scores ranged from −2.5 (95% CI −4.2, −0.8) for the body image domain to −7.7 (95% CI −14.7, −0.6) for the intimate relationships domain. For improvement, the mean LupusQoL change scores ranged from 5.6 (95% CI 4.2, 7.1) in the physical health domain to 10.4 (95% CI 7.7, 13.1) in the burden to others domain. The results for the SF‐36 were similar.

**Table 3:** Change in score of LupusQoL and SF‐36 in comparable domains by global rating of change category^a^

	Deterioration (−7 to −2)	Stable (−1 to 1)	Improvement (2 to 7)
LupusQoL			
Pain	−6.5 (−8.9, −4.1) [72, 195]	1.5 (0.2, 2.7) [89, 307]	9.3 (7.1, 11.5) [80, 258]
Physical health	−3.7 (−5.2, −2.1) [72, 188]	0.6 (−0.4, 1.6) [90, 298]	5.6 (4.2, 7.1) [83, 282]
Emotional health	−4.4 (−6.0, −2.7) [78, 213]	1.2 (0.1, 2.3) [92, 304]	6.2 (4.7, 7.8) [74, 243]
Fatigue	−4.6 (−6.5, −2.8) [81, 256]	2.1 (0.6, 3.6) [88, 296]	8.9 (6.8, 11.0) [62, 203]
SF‐36			
Bodily pain	−7.0 (−9.3, −4.7) [86, 254]	2.8 (1.1, 4.5) [80, 263]	13.0 (10.6, 15.4) [74, 231]
Physical functioning	−3.0 (−4.3, −1.6) [75, 209]	1.2 (0.3, 2.2) [89, 293]	5.6 (3.9, 7.3) [77, 249]
Mental health	−5.5 (−7.5, −3.6) [80, 234]	−0.1 (−1.6, 1.4) [84, 283]	7.6 (5.9, 9.4) [72, 238]
Vitality	−4.6 (−6.3, −2.8) [85, 286]	0.9 (−0.8, 2.6) [86, 283]	11.2 (8.4, 14.0) [62, 188]

a Values are mean (95% confidence interval) [number of contributing participants, number of valid change observations]. Totals = [101, 776]. SF‐36 = Short Form 36 health survey.

**Table 4:** Change in score of LupusQoL and SF‐36 in noncomparable domains by global rating of change category^a^

	Deterioration (−7 to −2)	Stable (−1 to 1)	Improvement (2 to 7)
LupusQoL			
Body image	−2.5 (−4.2, −0.8) [67, 197]	1.4 (0.3, 2.5) [79, 296]	6.4 (3.6, 9.2) [47, 122]
Planning	−4.6 (−7.0, −2.2) [64, 164]	1.2 (0.0, 2.4) [93, 391]	6.3 (3.9, 8.8) [65, 206]
Intimate relationships	−7.7 (−14.7, −0.6) [42, 75]	0.9 (−1.0, 2.8) [74, 338]	8.3 (4.3, 12.4) [45, 120]
Burden to others	−4.6 (−6.9, −2.3) [70, 195]	2.5 (1.2, 3.8) [94, 397]	10.4 (7.7, 13.1) [57, 167]
SF‐36			
General health	−2.0 (−3.2, −0.8) [84, 240]	0.3 (−0.6, 1.2) [87, 265]	3.4 (2.2, 4.6) [79, 259]
Role emotional	−10.1 (−15.9, −4.3) [69, 182]	2.6 (−0.3, 5.4) [89, 363]	11.3 (6.6, 15.9) [67, 204]
Role physical	−9.9 (−15.3, −4.5) [71, 185]	1.4 (−1.3, 4.0) [90, 339]	14.7 (9.9, 19.5) [72, 223]
Social functioning	−7.0 (−10.8, −3.1) [69, 178]	1.6 (0.1, 3.2) [92, 390]	10.1 (7.0, 13.2) [66, 190]

a Values are mean (95% confidence interval) [number of contributing participants, number of valid change observations]. Totals = [101, 776]. SF‐36 = Short Form 36 health survey.

When the overall disease activity lessened, 6 domains of the LupusQoL (physical health, pain, planning, emotional health, body image, and fatigue) and 7 of the SF‐36 domains (physical functioning, bodily pain, mental health, social functioning, role emotional, role physical, and vitality) showed an improvement. For the remaining LupusQoL and SF‐36 domains, changes were small and nonsignificant. When overall disease activity increased, there was no significant decrease in any of the scores of the LupusQoL or SF‐36 domains (see Supplementary Tables 2 and 3, available on the *Arthritis Care & Research* web site at http://onlinelibrary.wiley.com/doi/10.1002/acr.22850/abstract). An improvement in the disease activity of the musculoskeletal system was associated with significant improvements in both the LupusQoL and SF‐36 physical function and pain domains. When the disease activity worsened, only a significant deterioration of the LupusQoL pain domain was observed, although numbers of patients were low. When disease activity altered on the mucocutaneous system, no significant change was observed in the LupusQoL body image domain (see Supplementary Table 4, available on the *Arthritis Care & Research* web site at http://onlinelibrary.wiley.com/doi/10.1002/acr.22850/abstract).

For deterioration in GRC, LupusQoL domain SRMs ranged from −0.16 (body image) to −0.35 (pain), and those for SF‐36 ranged from −0.22 (general health) to −0.38 (bodily pain). For comparable domains, LupusQoL SRMs were similar in size to SF‐36 SRMs (see Supplementary Table 5, available on the *Arthritis Care & Research* web site at http://onlinelibrary.wiley.com/doi/10.1002/acr.22850/abstract). For improvement, LupusQoL domain SRMs ranged from 0.36 (planning and intimate relationships) to 0.55 (burden to others), and those for SF‐36 ranged from 0.25 (role emotional) to 0.61 (bodily pain). For comparable domains, LupusQoL and SF‐36 SRMs were similar in size. For effect sizes, patterns of sensitivity to change measures were similar (see Supplementary Table 6, available on the *Arthritis Care & Research* web site at http://onlinelibrary.wiley.com/doi/10.1002/acr.22850/abstract).

#### MID results

Using the anchor‐based approach, the MID(a)s for improvement and for deterioration for each of the LupusQoL and SF‐36 domains are given in Table 5. For deterioration, the mean MID(a) for the LupusQoL ranged from −2.4 (95% CI −4.8, 0.1) for body image to −8.7 (95% CI −18.9, 1.6) for intimate relationships, and for improvement from 3.5 (95% CI 0.5, 6.5) for body image to 7.3 (95% CI 4.0, 10.6) for burden to others. For the SF‐36, for deterioration, the mean MID(a) ranged from −2.0 (95% CI −3.4, −0.5) for general health to −11.1 (95% CI −17.8, −4.5) for role physical, and for improvement, the mean MID(a) ranged from 2.8 (95% CI 1.2, 4.5) for general health to 10.9 (95% CI 8.0, 13.8) for bodily pain and 10.9 (95% CI 7.5, 14.3) for vitality. For comparable domains, for both deterioration and improvement, mean MID(a)s for LupusQoL tended to be smaller in size than mean SF‐36 MID(a)s (Table 5).

**Table 5:** Minimal important change estimates for LupusQoL and SF‐36 in comparable and noncomparable domains via anchor‐based global rating of change category^a^

	Deterioration (−3 or −2)	Improvement (2 or 3)
Comparable domains		
LupusQoL pain	−4.7 (−7.6, −1.7)	6.8 (4.4, 9.1)
SF‐36 bodily pain	−6.7 (−9.4, −4.0)	10.9 (8.0, 13.8)
LupusQoL physical health	−3.4 (−5.1, −1.8)	4.0 (2.2, 5.8)
SF‐36 physical function	−2.4 (−4.3, −0.5)	3.8 (1.8, 5.8)
LupusQoL emotional health	−3.7 (−5.7, −1.7)	4.7 (2.6, 6.7)
SF‐36 mental health	−5.1 (−7.1, −3.2)	7.5 (5.3, 9.8)
LupusQoL fatigue	−3.2 (−5.4, −1.0)	6.6 (4.0, 9.1)
SF‐36 vitality	−3.5 (−5.5, −1.4)	10.9 (7.5, 14.3)
Noncomparable domains		
LupusQoL body image	−2.4 (−4.8, 0.1)	3.5 (0.5, 6.5)
LupusQoL planning	−4.0 (−7.4, −0.6)	3.8 (0.9, 6.6)
LupusQoL intimate relationships	−8.7 (−18.9, 1.6)	7.1 (2.1, 12.2)
LupusQoL burden to others	−5.0 (−7.8, −2.1)	7.3 (4.0, 10.6)
SF‐36 general health	−2.0 (−3.4, −0.5)	2.8 (1.2, 4.5)
SF‐36 role emotional	−10.4 (−18.1, −2.7)	10.2 (2.4, 18.0)
SF‐36 role physical	−11.1 (−17.8, −4.5)	10.8 (4.3, 17.4)
SF‐36 social functioning	−4.2 (−8.8, 0.3)	9.6 (5.4, 13.8)

a Values are mean (95% confidence interval). SF‐36 = Short Form 36 health survey.

Compared to anchor‐based approaches, using distribution‐based approaches based on 0.5 SD, LupusQoL domain MID(d)s were larger still, but more consistent between domains, ranging from 12.9 (emotional health) to 16.7 (intimate relationships). For SF‐36 domains, MID(d)s based on 0.5 SD were also larger, albeit relatively less so, ranging from 9.3 (general health perceptions) to 19.7 (role emotional). Estimates of MID(d) using 1 SEM were larger than anchor‐based estimates, ranging from 6.6 for emotional health to 13.2 for burden to others (see Supplementary Table 7, available on the *Arthritis Care & Research* web site at http://onlinelibrary.wiley.com/doi/10.1002/acr.22850/abstract).

## DISCUSSION

Knowing whether, or to what extent, a patient has improved or deteriorated following a course of treatment is fundamental to clinical practice. This work has demonstrated that all 8 of the LupusQoL domains are sensitive to change and able to identify patient‐reported improvements and deteriorations. With changes in physician‐reported disease activity, there were less consistent findings of improvement in 6 of 8 LupusQoL domains when disease activity lessened but little or no responsiveness with worsening disease activity. There may be several reasons for this difference: 1) physician‐reported disease activity measures a different concept from HRQOL, hence the FDA recommendation that responsiveness should be measured against the patient GRC, 2) patients may perceive improvement more clearly than deterioration, particularly after having a flare, 3) the number of patients in the deterioration subgroups, especially when single BILAG system changes were examined, may be insufficient to detect significant changes, and 4) the assessment over a month may be too short a time period for change to occur in some domains of the LupusQoL following a change in disease activity.

Different LupusQoL domains had different patient‐reported MID(a)s, which also differed for deterioration and improvement. When looking for an improvement in SLE, the MID ranges from 4 to 7 points, depending on the domain. For the SF‐36, the MIDs range from 3 to 11 points. These results will allow appropriate power calculations and interpretation of HRQOL measurements in clinical trials and longitudinal observational studies. MIDs are not without problems, in that different methodologies (anchor‐based or distribution‐based) generated somewhat different MIDs, and the MID reflects the difference that is important at a group, but not the individual, level. Regulatory bodies advocate the use of anchor‐based methods in the estimation of responsiveness as they use patient ratings 2, even though the reliability of patients' estimates of their previous health status has been questioned 30, 31.

This study recruited patients with moderate or severe flares and is likely to be representative of patients recruited into clinical trials. Notably, the original LupusQoL mean scores derived from consecutive outpatients at UK centers were strikingly higher (by approximately 20–25 points) across all domains 8 than the baseline values for these patients with moderate or severe flares. Such large differences suggest that a flare of SLE has a very significant impact on all aspects of HRQOL and may also explain why the LupusQoL is less responsive to deterioration of disease activity, as patients already have poor health.

There have been 2 publications regarding the sensitivity to change of the LupusQoL. Using the Canadian version of the LupusQoL, Touma et al 32 concluded that its responsiveness was similar to that of the SF‐36 following a 12‐month prospective cohort study of consecutive patients at a single tertiary center. However, only changes in the disease activity measure, the Systemic Lupus Erythematosus Disease Activity Index 2000 (SLEDAI‐2000) 33, 34, were used to estimate responsiveness, while in our study the patient‐reported GRC scale was used to estimate responsiveness as recommended by the regulatory bodies 2, 3, in addition to a disease activity measure (the BILAG‐2004 index). Results of a multitertiary center cohort study, recruiting consecutive patients using the French version of the LupusQoL, assessed patients at 3 and 6 months 35. The anchors for improvement and deterioration included a patient‐reported 7‐point Likert scale and visual analog scale (100 mm). A Likert scale of 5 patient‐reported symptoms extracted from the Systemic Lupus Assessment Questionnaire (SLAQ) was also used 36, 37. The French language LupusQoL and the SF‐36 showed comparable responsiveness, and the MIDs were similar for both measures. Despite the different patient selection criteria (consecutive recruitment/SLE flare; single tertiary center/multicenter study), length of followup period, different methods (anchor‐based, distribution‐based), and scales to evaluate sensitivity to change (GRC scale, SLAQ, SLEDAI‐2000), there is agreement that the LupusQoL demonstrates sensitivity to change in SLE.

In this study that recruited patients with active lupus, the LupusQoL and SF‐36 appear to be more responsive to improvement than deterioration; this result was also noted in the French study 35. Researchers previously reported that patients with other conditions detected improvements following treatment more easily than deterioration. Patients reported that they often did not realize how much they had deteriorated until they started to improve 38. This recognition is an encouraging finding, especially when the LupusQoL is recommended for use in clinical trials. When patients improve during and after an intervention, the LupusQoL should be able to detect these changes. In contrast, in a study of SLE patients that employed the SF‐36, deterioration of HRQOL was perceived more readily than improvement 39. However, that article described studies in a clinical trial setting, using an immunologic anchor as a marker of improvement.

In spite of a large data set and rigorous followup schedule, our study had little missing change score data on most domains (approximately 15%). The majority of patients were white (62.6%), but other groups were represented, including 15.2% of South Asian origin. Although monthly followups may not have allowed sufficient time for an intervention to take effect and for some HRQOL domains to change as different domains may change over different periods of time, monthly reviews did ensure that relapses and the effects of these on HRQOL were not missed.

The assessment of lupus disease in clinical trials should involve patient‐reported outcomes, including a global assessment and specific instruments that capture the impact of the disease on the patient quality of life. The LupusQoL has previously demonstrated good construct, face, discriminative, and concurrent validity, and internal and test–retest reliability, and has been mapped to the Short Form in 6 dimensions 8, 40, 41. Linguistic validations have enabled the instrument to be employed successfully in 51 countries using 77 different languages 42. This study demonstrates the responsiveness of the instrument and further construct validity as compared with the SF‐36 and provides the MIDs. The SF‐36 and the LupusQoL are similar in terms of responsiveness, but the items on the LupusQoL were informed by patients with SLE, and therefore it has the advantage of several SLE‐specific domains that are important to patients (planning, burden to others, intimate relationships, and body image) 9 that are not captured by the SF‐36.

## AUTHOR CONTRIBUTIONS

All authors were involved in drafting the article or revising it critically for important intellectual content, and all authors approved the final version to be submitted for publication. Dr. Teh had full access to all of the data in the study and takes responsibility for the integrity of the data and the accuracy of the data analysis.

### Study conception and design

McElhone, Abbott, Sutton, Gordon, Teh.

### Acquisition of data

McElhone, Abbott, Lanyon, Rahman, Yee, Akil, Bruce, Ahmad, Gordon, Teh.

### Analysis and interpretation of data

McElhone, Abbott, Sutton, Mullen, Gordon, Teh.

## Supporting information

Supplementary Table 1 – Disease Activity as assessed by BILAG‐2004, n (%) unless stated
Supplementary Table 2: LupusQoL and SF‐36 mean (with 95%CI) change of score for BILAG change (broad categorisation) [number of contributing participants; number of valid change observations] for comparable domains
Supplementary Table 3: LupusQoL and SF‐36 mean (with 95%CI) change of score for BILAG change (broad categorisation) [number of contributing participants; number of valid change observations] for non‐comparable domains
Supplementary Table 4: Mean (with 95% Confidence interval) change of score of LupusQoL Physical health and Pain domains and SF‐36 Physical functioning and bodily pain domains compared to broad disease activity change of the musculoskeletal system of the BILAG‐2004 index and of the LupusQoL Body Image domain to the mucocutaneous system of the BILAG‐2004 index ([number of contributing participants; number of valid change observations]
Supplementary Table 5: Responsiveness (SRM) of LupusQoL and SF‐36 in comparable and non‐comparable domains by Global Rating of Change category
Supplementary Table 6: Responsiveness (ESs) of LupusQoL and SF‐36 in comparable and non‐comparable domains by Global Rating of Change category
Supplementary Table 7: Minimal important change estimates for LupusQoL and SF‐36 in comparable and non‐comparable domains via anchor‐based and distribution‐based criteria by Global Rating of Change category
