# Supplementary material for: Sensitivity to Change and Minimal Important Differences of the LupusQoL in Patients With Systemic Lupus Erythematosus
Source: Arthritis Care Res (Hoboken). 2016 Sep 2;68(10):1505–13. doi: 10.1002/acr.22850 (PMC5053261; doi:10.1002/acr.22850)
Supplement: Supplementary file 1 — Supplementary Table 1 – Disease Activity as assessed by BILAG‐2004, n (%) unless stated Supplementary Table 2: LupusQoL and SF‐36 mean (with 95%CI) change of score for BILAG change (broad categorisation) [number of contributing participants; number of valid change observations] for comparable domains Supplementary Table 3: LupusQoL and SF‐36 mean (with 95%CI) change of score for BILAG change (broad categorisation) [number of contributing participants; number of valid change observations] for non‐comparable domains Supplementary Table 4: Mean (with 95% Confidence interval) change of score of LupusQoL Physical health and Pain domains and SF‐36 Physical functioning and bodily pain domains compared to broad disease activity change of the musculoskeletal system of the BILAG‐2004 index and of the LupusQoL Body Image domain to the mucocutaneous system of the BILAG‐2004 index ([number of contributing participants; number of valid change observations] Supplementary Table 5: Responsiveness (SRM) of LupusQoL and SF‐36 in comparable and non‐comparable domains by Global Rating of Change category Supplementary Table 6: Responsiveness (ESs) of LupusQoL and SF‐36 in comparable and non‐comparable domains by Global Rating of Change category Supplementary Table 7: Minimal important change estimates for LupusQoL and SF‐36 in comparable and non‐comparable domains via anchor‐based and distribution‐based criteria by Global Rating of Change category [file ACR-68-1505-s001.docx]

**Supplementary Table 1 – Disease Activity as assessed by BILAG-2004, n (%) unless stated**

| BILAG-2004: Index Mean (SD)    A score in one or more systems  Constitutional  A  B  Mucocutaneous  A  B  Neuropsychiatric  A  B  Musculoskeletal  A  B  Cardiorespiratory  A  B  Gastrointestinal  A  B  Ophthalmic  A  B  Renal  A  B  Haematology  A  B | 16.4 (8.1)  41 (41%)  2 (2%)  11 (11%)  6 (6%)  33 (33%)  1 (1%)  3 (3%)  28 (28%)  39 (39%)  0 (0%)  23 (23%)  0 (0%)  4 (4%)  0 (0%)  2 (2%)  5 (5.0%)  15 (15%)  0 (0%)  0 (0%) |
| --- | --- |

**Supplementary Table 2: LupusQoL and SF-36 mean (with 95%CI) change of score for BILAG change (broad categorisation) [number of contributing participants; number of valid change observations] for comparable domains**

|  | Comparable domains | | | | | | | |
| --- | --- | --- | --- | --- | --- | --- | --- | --- |
| BILAG-2004 broad change category | LupusQoL  Physical health | SF-36  Physical functioning | LupusQoL  Pain | SF-36  Bodily pain | LupusQoL  Emotional health | SF-36  Mental health | LupusQoL  Fatigue | SF-36  Vitality |
| Deterioration | -0.8  (-3.0 to 1.4)  [73;155] | 0.3  (-1.8 to 2.4) [72;154] | -2.4  (-5.9 to 1.1)  [71;152] | -2.2  (-6.0 to 1.7) [73;154] | -1.1  (-3.5 to 1.3)  [73;155] | -2.4  (-5.2 to 0.4) [73;154] | -1.0  (-3.5 to 1.5)  [71;152] | -2.0  (-4.8 to 0.8) [73;154] |
| Improvement | 4.0  (1.9 to 6.1)  [96;187] | 3.7  (1.3 to 6.0) [95;185] | 7.7  (4.8 to 10.5)  [96;185] | 8.2  (5.2 to 11.3) [95;186] | 3.1  (0.8 to 5.4)  [95;183] | 3.8  (1.3 to 6.2) [94;184] | 4.1  (1.7 to 6.5)  [95;182] | 5.5  (2.5 to 8.5) [94;184] |
| Persistent active disease | -0.9  (-2.7 to 0.9)  [54;120] | -1.1  (-2.9 to 0.7) [54;118] | -0.9  (-3.6 to 1.7)  [53;119] | 0.9  (-1.4 to 3.1) [54;119] | 0.3  (-1.7 to 2.3)  [53;117] | -0.3  (-2.6 to 1.9) [54;119] | 2.0  (-0.7 to 4.7)  [53;115] | -0.6  (-3.2 to 2.0) [54;119] |
| Persistent inactive disease | 1.6  (0.6 to 2.7)  [65;235] | 1.7  (0.5 to 3.0) [65;231] | 2.0  (0.5 to 3.5)  [65;232] | 2.7  (0.7 to 4.7) [65;235] | 2.3  (1.1 to 3.5)  [65;233] | 1.2  (-0.3 to 2.7) [65;234] | 1.7  (0.2 to 3.2)  [65;231] | 1.7  (0.1 to 3.3) [65;234] |

**Supplementary Table 3: LupusQoL and SF-36 mean (with 95%CI) change of score for BILAG change (broad categorisation) [number of contributing participants; number of valid change observations] for non-comparable domains**

|  | Non-comparable domains | | | | | | | |
| --- | --- | --- | --- | --- | --- | --- | --- | --- |
| BILAG-2004 global change category | LupusQoL  Planning | LupusQoL  Intimate relationships | LupusQoL  Burden to others | LupusQoL  Body Image | SF-36  Social functioning | SF-36  General health | SF-36  Role emotional | SF-36  Role physical |
| Deterioration | -1.2  (-3.8 to 1.4)  [72;153] | 2.6  (-1.3 to 6.5)  [54;110] | 2.3  (-0.6 to 5.2)  [73;151] | 0.5  (-2.2 to 3.2)  [64;136] | -2.6  (-5.8 to 0.7) [73;155] | -0.6  (-2.0 to 0.8) [73;155] | -0.9  (-6.6 to 4.9) [73;152] | -4.4  (-9.0 to 0.3) [73;151] |
| Improvement | 2.6  (0.1 to 5.1)  [96;185] | 2.4  (-1.2 to 6.0)  [75;140] | 2.4  (-0.1 to 4.8)  [96;186] | 3.1  (0.1 to 6.1)  [75;145] | 4.5  (1.5 to 7.4) [95;185] | 1.3  (-0.2 to 2.9) [95;185] | 6.4  (0.4 to 12.3) [95;183] | 4.9  (0.4 to 9.5) [95;184] |
| Persistent active disease | 0.0  (-1.9 to 1.8)  [54;120] | -2.5  (-6.3 to 1.3)  [42;90] | -1.2  (-4.0 to 1.6)  [54;120] | 0.6  (-2.0 to 3.2)  [45;97] | -0.3  (-3.2 to 2.6) [54;120] | -0.3  (-1.5 to 0.8) [54;120] | 0.6  (-4.6 to 5.7) [54;119] | 2.4  (-1.5 to 6.3) [53;117] |
| Persistent inactive disease | 2.5  (0.9 to 4.0)  [65;231] | 1.8  (-0.8 to 4.5)  [46;143] | 4.1  (2.2 to 6.0)  [63;233] | 0.2  (-1.2 to 1.5)  [53;173] | 3.2  (1.1 to 5.4) [65;235] | 1.4  (0.5 to 2.4) [65;235] | 3.3  (0.4 to 6.2) [65;233] | 5.9  (2.7 to 9.0) [65;233] |

**Supplementary Table 4: Mean (with 95% Confidence interval) change of score of LupusQoL Physical health and Pain domains and SF-36 Physical functioning and bodily pain domains compared to broad disease activity change of the musculoskeletal system of the BILAG-2004 index and of the LupusQoL Body Image domain to the mucocutaneous system of the BILAG-2004 index ([number of contributing participants; number of valid change observations]**

| BILAG-2004 change category | LupusQoL Physical Health  (for BILAG-2004 musculoskeletal system) | SF-36 Physical Functioning  (for BILAG-2004 musculoskeletal system) | LupusQoL Pain  (for BILAG-2004 musculoskeletal system) | SF-36 Bodily Pain  (for BILAG-2004 musculoskeletal system) | LupusQoL Body Image  (for BILAG-2004 mucocutaneous system) |
| --- | --- | --- | --- | --- | --- |
| Deterioration | -3.7 (-7.8 to 0.3)  [41;53] | -2.3 (-6.2 to 1.5)  [41;53] | -7.0 (-12.8 to -1.2)  [40;51] | -6.0 (-12.4 to 0.4)  [41;53] | -1.1 (-4.4 to 2.3)  [35;48] |
| Improvement | 3.5 (0.5 to 6.5)  [69;113] | 5.9 (3.0 to 8.8)  [69;113] | 8.0 (3.7 to 12.2)  [69;111] | 9.9 (6.0 to 13.7)  [69;113] | 3.6 (-0.4 to 7.6)  [47;77] |
| Persistent active disease | 0.2 (-2.5 to 3.0)  [33;73] | -1.7 (-4.7 to 1.3)  [33;73] | 1.4 (-3.2 to 6.1)  [32;72] | 3.5 (0.1 to 6.9)  [33 73] | -0.8 (-4.8 to 3.2)  [26;46] |
| Persistent inactive disease | 1.5 (0.8 to 2.2)  [89;458] | 1.3 (0.5 to 2.1)  [89;449] | 1.7 (0.8 to 2.6)  [89;454] | 1.9 (0.8 to 3.1)  [89;455] | - 1. (0.0 to 2.2)   [77;380] |

**Supplementary Table 5: Responsiveness (SRM) of LupusQoL and SF-36 in comparable and non-comparable domains by Global Rating of Change category**

| Comparable Domains  SRM Change from Previous Visit | | | | | | | | |
| --- | --- | --- | --- | --- | --- | --- | --- | --- |
|  | LupusQoL  Pain | SF-36  Bodily Pain | LupusQoL  Physical Health | SF-36  Physical Functioning | LupusQoL  Emotional Health | SF-36  Mental Health | LupusQoL  Fatigue | SF-36  Vitality |
| Deterioration | -0.35 | -0.38 | -0.30 | -0.24 | -0.29 | -0.34 | -0.32 | -0.26 |
| Stable | 0.11 | 0.17 | 0.06 | 0.11 | 0.1 | -0.01 | 0.16 | 0.05 |
| Improvement | 0.49 | 0.61 | 0.41 | 0.37 | 0.42 | 0.52 | 0.48 | 0.59 |
| Non-comparable Domains  SRM Change from Previous Visit | | | | | | | | |
|  | LupusQoL  Body Image | LupusQoL  Planning | LupusQoL  Intimate Relationships | LupusQoL  Burden to others | SF-36  General Health | SF-36  Role Emotional | SF-36  Role Physical | SF-36  Social Functioning |
| Deterioration | -0.16 | -0.27 | -0.27 | -0.26 | -0.22 | -0.28 | -0.35 | -0.29 |
| Stable | 0.11 | 0.09 | 0.05 | 0.16 | 0.04 | 0.07 | 0.04 | 0.08 |
| Improvement | 0.40 | 0.36 | 0.36 | 0.55 | 0.29 | 0.25 | 0.38 | 0.46 |

**Supplementary Table 6: Responsiveness (ESs) of LupusQoL and SF-36 in comparable and non-comparable domains by Global Rating of Change category**

| Comparable Domains  Effect Size | | | | | | | | |
| --- | --- | --- | --- | --- | --- | --- | --- | --- |
|  | Lupus Qol  Pain | SF-36  Bodily Pain | Lupus Qol  Physical Health | SF-36 Physical Functioning | Lupus Qol  Emotional Health | SF-36  Mental Health | Lupus Qol  Fatigue | SF-36  Vitality |
| Deterioration | -0.24 | -0.31 | -0.13 | -0.11 | -0.16 | -0.25 | -0.17 | -0.21 |
| Stable | 0.04 | 0.10 | 0.02 | 0.04 | 0.04 | 0.00 | 0.07 | 0.04 |
| Improvement | 0.36 | 0.53 | 0.22 | 0.20 | 0.26 | 0.38 | 0.39 | 0.50 |

| Non-Comparable Domains  Effect Size | | | | | | | | |
| --- | --- | --- | --- | --- | --- | --- | --- | --- |
|  | Lupus Qol  Body Image | Lupus Qol  Planning | Lupus Qol  Intimate Relationships | Lupus Qol  Burden to Others | SF-36  General Health | SF-36  Role Emotional | SF-36  Role Physical | SF-36 Social Functioning |
| Deterioration | -0.09 | -0.16 | -0.24 | -0.16 | -0.12 | -0.25 | -0.30 | -0.28 |
| Stable | 0.05 | 0.03 | 0.02 | 0.07 | 0.01 | 0.06 | 0.03 | 0.05 |
| Improvement | 0.27 | 0.22 | 0.26 | 0.37 | 0.17 | 0.26 | 0.38 | 0.40 |

**Supplementary Table 7: Minimal important change estimates for LupusQoL and SF-36 in comparable and non-comparable domains via anchor-based and distribution-based criteria by Global Rating of Change category**

| Comparable Domains | | | | | | | | |
| --- | --- | --- | --- | --- | --- | --- | --- | --- |
| Minimal important change estimate | LupusQoL  Pain | SF-36  Bodily Pain | LupusQoL  Physical Health | SF-36  Physical Functioning | LupusQoL  Emotional Health | SF-36  Mental Health | LupusQoL  Fatigue | SF-36  Vitality |
| Mean (95%CI) anchor-based minimal important deterioration (GRC: -3 or -2) [number of contributing participants; number of change observations] | -4.7  (-7.6 to -1.7)  [60;128 ] | -6.7  (-9.4 to -4.0) [77;172] | -3.4  (-5.1 to -1.8)  [56;117 ] | -2.4  (-4.3 to -0.5) [64;134] | -3.7  (-5.7 to -1.7)  [61;134] | -5.1  (-7.1 to -3.2) [70;153] | -3.2  (-5.4 to -1.0)  [70;163] | -3.5  (-5.5 to -1.4) [78;84] |
| Mean (95%CI) anchor-based minimal important improvement (GRC: +2 or +3) [number of contributing participants; number of change observations] | 6.8  (4.4 to 9.1)  [69;158] | 10.9  (8.0 to 13.8) [65;156] | 4.0  (2.2 to 5.8)  [72;179] | 3.8  (1.8 to 5.8) [67;159] | 4.7  (2.6 to 6.7)  [62;138] | 7.5  (5.3 to 9.8) [63;149] | 6.6  (4.0 to 9.1)  [52;122] | 10.9  (7.5 to 14.3) [55;115] |
| Distribution-based minimal important change: 1 SEM* | 8.0 | - | 7.1 | - | 6.6 | - | 11.2 | - |
| Distribution-based minimal important change: 0.5 SD | 14.0 | 11.6 | 13.3 | 13.4 | 12.9 | 10.3 | 13.0 | 10.0 |
| Non-Comparable Domains | | | | | | | | |
|  | LupusQoL  Body Image | LupusQoL  Planning | LupusQoL  Intimate Relationships | LupusQoL  Burden to others | SF-36  General Health | SF-36  Role Emotional | SF-36  Role Physical | SF-36  Social Functioning |
| Mean (95%CI) anchor-based minimal important deterioration (GRC: -3 or -2) [number of contributing participants; number of change observations] | -2.4  (-4.8 to 0.1)  [58;123] | -4.0  (-7.4 to -0.6)  [52;96] | -8.7  (-18.9 to 1.6)  [31;49] | -5.0  (-7.8 to -2.1)  [58;126] | -2.0  (-3.4 to -0.5) [71;159] | -10.4  (-18.1 to -2.7) [59;119] | -11.1  (-17.8 to -4.5) [61;122] | -4.2  (-8.8 to 0.3) [61;110] |
| Mean (95%CI) anchor-based minimal important improvement (GRC change: +2 or +3) [number of contributing participants; number of change observations] | 3.5  (0.5 to 6.5)  [40;76] | 3.8  (0.9 to 6.6)  [52;121] | 7.1  (2.1 to 12.2)  [32;63] | 7.3  (4.0 to 10.6)  [45;88] | 2.8  (1.2 to 4.5) [72;177] | 10.2  (2.4 to 18.0) [55; 124] | 10.8  (4.3 to 17.4) [65;143] | 9.6  (5.4 to 13.8) [57;122] |
| Distribution-based minimal important change: 1 SEM* | 8.2 | 10.5 | - | 13.2 | - | - | - | - |
| Distribution-based minimal important change: 0.5 SD | 14.5 | 16.1 | 16.7 | 14.1 | 9.3 | 19.7 | 13.8 | 14.9 |

* From LupusQoL test-retest data as presented as reliability coefficients in McElhone *et al* (8)*,* where available
